# The effect of repeated bouts of electrical stimulation‐induced muscle contractions on proteolytic signaling in rat skeletal muscle

**DOI:** 10.14814/phy2.14842

**Published:** 2021-05-15

**Authors:** Takaya Kotani, Junya Takegaki, Yuki Tamura, Karina Kouzaki, Koichi Nakazato, Naokata Ishii

**Affiliations:** ^1^ Department of Life Sciences Graduate School of Arts and Sciences The University of Tokyo Tokyo Japan; ^2^ Graduate School of Health and Sport Science Nippon Sport Science University Tokyo Japan; ^3^ Ritsumeikan Global Innovation Research Organization Ritsumeikan University Shiga Japan

**Keywords:** calpain, LC3, mTORC1, muscle protein breakdown, resistance exercise, ubiquitinated protein

## Abstract

Mechanistic target of rapamycin complex 1 (mTORC1) plays a central role in muscle protein synthesis and repeated bouts of resistance exercise (RE) blunt mTORC1 activation. However, the changes in the proteolytic signaling when recurrent RE bouts attenuate mTORC1 activation are unclear. Using a RE model of electrically stimulated rat skeletal muscle, this study aimed to clarify the effect of repeated RE bouts on acute proteolytic signaling, particularly the calpain, autophagy‐lysosome, and ubiquitin‐proteasome pathway. p70S6K and rpS6 phosphorylation, indicators of mTORC1 activity, were attenuated by repeated RE bouts. Calpain 3 protein was decreased at 6 h post‐RE in all exercised groups regardless of the bout number. Microtubule‐associated protein 1 light chain 3 beta‐II, an indicator of autophagosome formation, was increased at 3 h and repeated RE bouts increased at 6 h, post‐RE. Ubiquitinated proteins were increased following RE, but these increases were independent of the number of RE bouts. These results suggest that the magnitude of autophagosome formation was increased following RE when mTORC1 activity was attenuated with repeated bouts of RE.

## Graphical abstract

Repeated bouts of resistance exercise (RE) attenuate the degree of exercise‐induced mechanistic target of rapamycin complex 1 (mTORC1) activation in skeletal muscle. The present study suggests that repeated bouts of RE increased light chain 3 beta (LC3)‐II protein (an indicator of autophagosome formation) following RE when mTORC1 activation was attenuated with repeated bouts of RE in rat skeletal muscle.

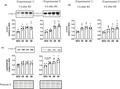

## 1 INTRODUCTION

Skeletal muscle is a highly plastic tissue, and skeletal muscle mass is determined by the net balance of muscle protein synthesis and muscle protein breakdown. Resistance exercise (RE) accelerates both muscle protein synthesis and breakdown, and the balance shifts toward net protein synthesis (Phillips et al., 1997). RE activates the mechanistic target of rapamycin complex 1 (mTORC1) signaling, which regulates muscle protein synthesis through the translation initiation in the ribosomes (Baar & Esser, 1999; Bodine, Latres, et al., 2001; Bodine, Stitt, et al., 2001; Burd et al., 2010; Drummond et al., 2009; Kubica et al., 2005; Nader et al., 2005). Repeated bouts of RE attenuate the mTORC1 signaling response to RE (Coffey et al., 2005; Ogasawara et al., 2013), which leads to a decreased degree of muscle protein synthesis activation and the muscle hypertrophy rate. However, mTORC1 negatively regulates the proteolytic response. Thus, it is possible that attenuation of mTORC1 activity with repeated bouts of RE induces not only a reduction in muscle protein synthesis but also an increase in muscle protein breakdown. However, it is unclear whether the activation of the proteolytic response is changed when mTORC1 activity is physiologically attenuated with repeated bouts of RE.

Recently, it has been suggested that muscle protein breakdown is also important for muscle remodeling and RE adaptation (Bell et al., 2016; Pasiakos, & Carbone, 2014). The proteolytic signals are known to be involved in the muscle protein degradation system after exercise: the calpain pathway, the autophagy–lysosome pathway, and the ubiquitin‐proteasome pathway. The calpain pathway involves calcium‐activated proteases and is important in cytoskeletal structural remodeling in skeletal muscle. Previous studies have reported that calpain gene expression was changed after acute RE in human skeletal muscle (Deldicque et al., 2008; Féasson et al., 2002); however, little is known about the effect of repeated bouts of RE. In the autophagy–lysosome system, the 5′ AMP‐activated protein kinase activates the UNC‐51‐like kinase (ULK1), thereby forming the isolation membrane and autophagosome (Li et al., 2013) and degrading the lysosome that binds to the autophagosome (Mizushima et al., 2011). The microtubule‐associated protein 1 light chain 3 beta (LC3)‐II correlates with autophagosome content, making it an indicator of autophagosome formation (Mizushima, & Yoshimori, 2007). mTORC1 signaling negatively regulates the autophagy‐lysosome system via ULK1 (Ser‐757) phosphorylation (Kim et al., 2011). Chronic pharmacological inhibition of mTORC1 increases LC3 expression in rat skeletal muscle (Ogasawara et al., 2016). The ubiquitin–proteasome system is also associated with muscle protein breakdown (Bodine, & Baehr, 2014; Bodine, Latres, et al., 2001; Bodine, Stitt, et al., 2001). The two muscle‐specific E3 ubiquitin ligases, the muscle‐specific RING finger 1 (MuRF‐1), and the muscle atrophy F‐box (MAFbx, also known as atrogin‐1), have been identified in skeletal muscle (Bodine, Latres, et al., 2001; Bodine, Stitt, et al., 2001). The target proteins are polyubiquitinated by these ubiquitin ligases and subsequently degraded by the 26S proteasome. The ubiquitin–proteasome system is also negatively regulated by mTORC1 signaling (Zhao et al., 2015).

The effects of repeated bouts of RE on the proteolytic response have been examined in previous studies. Mascher et al. (2008) reported that the second session of RE has a lower expression of MuRF‐1 messenger RNA (mRNA) in human skeletal muscle than the first session 2 h after exercise. However, Nader et al. (2014) reported that LC3, MuRF‐1, and Atrogin‐1 mRNA expressions following exercise on the 24th RE did not change compared with the first RE 4 h after exercise. There is no consensus on the effect of repeated bouts of RE on ubiquitin ligase expression in the previous studies. Furthermore, the effect of repeated bouts of RE on ubiquitinated protein and LC3 protein when mTORC1 activity is physiologically attenuated remains unknown.

Our group created a rat RE model with electrical stimulation‐induced muscle contraction and reported that repeated bouts of RE of every other day or three times a week induce muscle hypertrophy (Kotani et al., 2019; Ogasawara et al., 2013, 2014). Using this RE model with rat skeletal muscle, we examined the effects of repeated RE bouts on the acute proteolytic signaling responses involved in both the autophagy–lysosome and ubiquitin–proteasome systems to obtain an insight into the proteolytic response with repeated bouts of RE. We hypothesized that repeated bouts of RE would induce an increase in the formation of autophagosome and ubiquitination, contrary to the attenuation of mTORC1 activity.

## 2 METHODS

### 2.1 Ethical approval

This study was approved by the Animal Ethics and Research Committee of The University of Tokyo (no. 27‐13), and all experiments followed the guidelines of the committee. All experiments were performed in accordance with the guidelines of the Animal Ethics and Research Committee of The University of Tokyo and conformed to the principles and standards for reporting animal experiments of the Journal of Physiology and Physiological Reports.

### 2.2 Experimental animals

**FIGURE 1:**
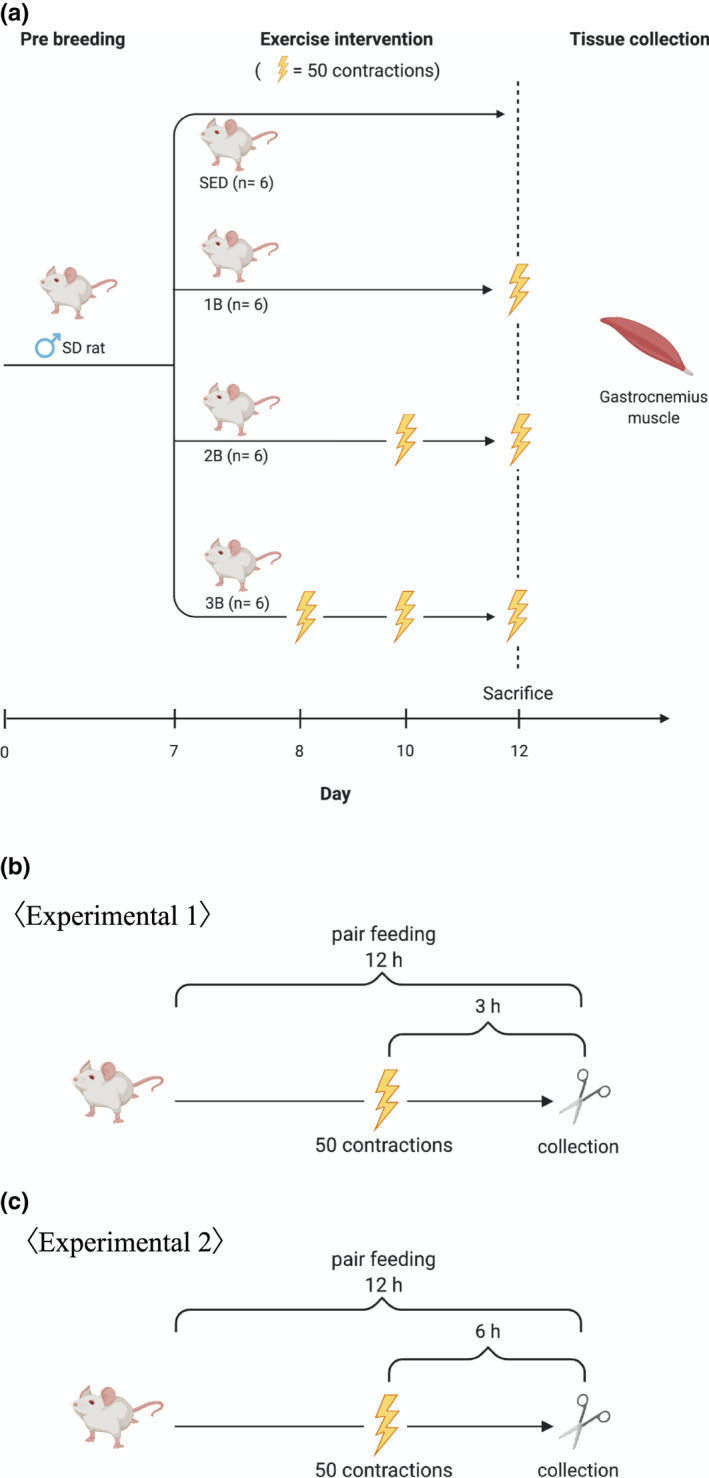
Experimental design. (a) SED, sedentary for 5 days; 1B, 2B, and 3B indicate resistance exercised with one bout, two bouts, three bouts (*n* = 6 for each group). (b) Muscle samplings were carried out 3 (Experimental 1) and 6 h (Experimental 2) after the last resistance exercise

Forty‐two male Sprague–Dawley rats (weight, 300–320 g) aged 10‐week‐old were obtained from SLC Japan. All animals were housed for 1 week in an environmentally controlled room at 23°C–25°C on a 12:12‐h light–dark cycle with free access to normal food (CE‐7; Clea Japan) and water. As shown in Figure 1, the rats were randomly assigned to the following seven groups (*n* = 6 for each group): sedentary for 5 days (SED), resistance‐exercise trained with one bout (1B), two bouts (2B), and three bouts (3B). Previous studies observed the RE‐induced peak activation of mTORC1 and proteolytic responses 3–6 h after exercise (Ogasawara et al., 2014; Phillips et al., 1997; West et al., 2016). Using isoflurane deep sedation, rats were killed by exsanguination after collection of whole blood from the abdominal aorta after 12 h of pair feeding, and muscle sampling was performed at 3 h (Experiment 1) and 6 h (Experiment 2) after RE (Figure 1B,C). Collected muscle samples were frozen at −80°C.

### 2.3 Resistance exercise

The RE protocol was carried out as previously described (Kotani et al., 2019). Briefly, with rats under 3.5%–4.0% isoflurane deep sedation, we shaved the hair on the right leg of each rat off, and then the rat was secured by its right foot on a footplate in the prone posture. The ankle joint was positioned at an angle of 0° (90° relative to the tibia). The triceps surae muscle was stimulated percutaneously with disposable electrodes (Vitrode V, Ag/AgCl; Nihon Kohden) connected to an electrical stimulator and isolator (SS‐104J; Nihon Kohden). The right gastrocnemius muscle was isometrically exercised with five sets of isometric contractions (3‐sec stimulation × 10 contractions, with 7‐s intervals between contractions, per set, with a 3‐min rest interval). The voltage (~30 V) and stimulation frequency (100 Hz) were adjusted to evoke maximal isometric contraction (Ogasawara et al., 2014). The frequency of RE bouts was every other day (48 h between bouts) in the 2B and 3B groups because previous studies showed that this frequency induces muscle hypertrophy in greater than or equal to12B (Kotani et al., 2019; Ogasawara et al., 2014, 2016).

### 2.4 Western blot analysis

Western blot analysis was performed as reported previously (Kotani et al., 2019). Briefly, frozen muscle samples were homogenized in a RIPA buffer with a protease and phosphatase inhibitor cocktail (catalog no. 15624189; Thermo Fisher Scientific). Homogenates were centrifuged at 10,000 *g* for 10 min at 4°C, after which the supernatants were collected. Protein concentrations in the supernatant were determined using a Protein Assay II kit (Bio‐Rad). Samples were diluted in 3× Blue Loading Buffer (catalog no. 7722; Cell Signaling Technology) and boiled at 95°C for 5 min. Further, 20 µg of proteins were separated on 7–15% SDS–polyacrylamide gels by electrophoresis and subsequently transferred to a polyvinylidene difluoride membrane. Membranes were stained with Ponceau S (catalog no. 33427.01; Serving Scientists) and scanned for protein normalization with a chemiluminescence detector (ChemiDoc MP; Bio‐Rad). Each membrane was washed in Tris‐buffered saline containing 0.1% Tween‐20 (TBST) and then blocked with 5% bovine serum albumin in TBST for 1 h at room temperature. After washing, the membrane was incubated overnight at 4°C with 3% bovine serum albumin in TBST and primary antibodies: phosphorylated (phospho)‐extracellular signal‐regulated kinase (ERK) (Tyr202/Thr204, 1:1000, catalog no. 9101), total ERK (1:1000, catalog no. 9102), phospho‐tuberous sclerosis complex 2 (TSC2; Ser664, 1:1000, catalog no. 40729), total TSC2 (1:1000, catalog no. 4308), phospho‐protein kinase B (Akt; Ser473, 1:1000, catalog no. 4051), total Akt (1:1000, catalog no. 9272), phospho‐TSC2 (Ser939, 1:1000, catalog no. 3615), phospho‐TSC2 (Ser1462, 1:1000, catalog no. 3617), phospho‐p70S6K (Thr389, 1:1000, catalog no. 9205), total p70S6K (1:1000, catalog no. 2708), phospho‐rpS6 (Ser240/244, 1:1000, catalog no. 2215), total rpS6 (1:1000, catalog no. 2217), phospho‐TSC2 (Ser1387, 1:1000, catalog no. 5582), total LC3 (1:1000, catalog no. 2775), phospho‐ULK1 (Ser555, 1:1000, catalog no. 5869), phospho‐ULK1 (Ser757, 1:1000, catalog no. 14202), total ULK1 (1:1000, catalog no. 8054), phospho‐forkhead box O3a (Foxo3a; Ser253, 1:1000, catalog no. 9466), total Foxo3a (1:1000, catalog no. 2497), ubiquitin (1:1000, catalog no. 3933) were purchased from Cell Signal Technology. Total p62/SQSTM1 (1:1000, catalog no. PM045) was obtained from Medical & Biological Laboratories. Calpain 1 (1:1000, catalog no. 10538‐1‐AP), Calpain 2 (1:1000, catalog no. 11472‐1‐AP), and Calpain 3 (1:1000, catalog no. 28476‐1‐AP) were purchased from proteintech. Total Atrogin‐1 (1:1000, catalog no. ab168372) and total MuRF‐1 (1:1000, catalog no. ab172479) were purchased from Abcam. The membranes were washed in TBST and incubated for 1 h at room temperature with appropriate secondary antibodies. A chemiluminescence reagent (Clarity Western ECL Substrate; Bio‐Rad) was used to visualise protein bands. Images were scanned with a chemiluminescence detector (ChemiDoc MP; Bio‐Rad). Band intensity was quantified using Image Lab v.5.2.1 software (Bio‐Rad). Ubiquitinated protein was calculated for the whole lane. We normalized protein bands with Ponceau bands.

### 2.5 Real‐time quantitative polymerase chain reaction

A real‐time quantitative polymerase chain reaction (RT‐qPCR) was performed as previously described (Kotani et al., 2019). Total RNA was extracted from a frozen muscle sample (the same gastrocnemius used for protein extractions) using TRIzol^TM^ LS Reagent (catalog no. 10296028; Thermo Fishier Scientific). The total RNA concentration was measured using a spectrophotometer (Nano Drop^TM^ 1000; Thermo Fisher Scientific). Complement DNA (cDNA) was synthesized from 1 µg total RNA using a high‐capacity cDNA reverse transcription kit (Applied Biosystems). Gene expression levels were quantified using THUNDERBIRD SYBR qPCR Mix (Toyobo) on a real‐time polymerase chain reaction system (CFX96; Bio‐Rad) under the following conditions: 95°C for 30 s, followed by 40 cycles at 95°C for 5 s and 60°C for 1 min. Primers for RT‐qPCR were obtained from Eurofins. The following primer sequences were used: MuRF‐1; forward 5′‐GACATCTTCCAGGCTG‐3′, MuRF‐1; reverse 5′‐TGCCGGTCCATGATCA‐3′, Atrogin‐1; forward 5′‐AGCTCCAACAGCCTTA‐3′, Atrogin‐1; reverse 5′‐AAGGAGCGCCATGGAT‐3′, glyceraldehyde 3‐phosphate dehydrogenase (GAPDH); forward 5′‐GTCGTGGAGTCTACTGGCGTCTT‐3′, GAPDH; and reverse 5′‐CAGTCTTCTGAGTGGCAGTGATGG‐3′. We observed no significant differences in GAPDH mRNA expressions between values at any time. Therefore, GAPDH was used for normalization.

### 2.6 Data analysis

Data are presented as mean ± SD and were analyzed using SPSS v.24.0 (IBM). Differences between groups were evaluated by one‐way ANOVA with Tukey's post hoc test. Statistical significance was set at *p* < 0.05.

## 3 RESULTS

### 3.1 Upstream‐regulatory factors of mTORC1 signaling

**FIGURE 2:**
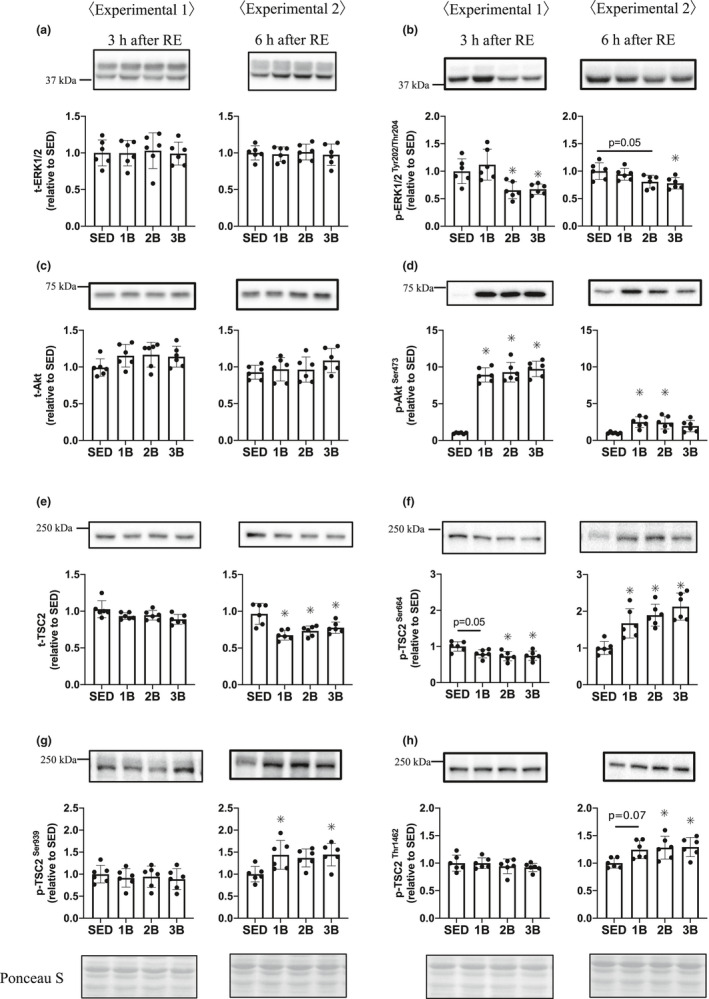
Effects of the number of resistance exercise (RE) bouts on extracellular signal‐regulated kinase (ERK; a, b), Akt (c, d), and TSC2 (e–h). Protein phosphorylation and content are expressed relative to the mean value in the SED group. 1B, 2B, and 3B indicate resistance exercised with one bout, two bouts, and three bouts, respectively (*n* = 6 for each group). The timing of muscle sampling is denoted above each diagram. Values in the data are means ± SD. SED, sedentary for 5 days. *p < 0.05 versus SED

Extracellular signal‐regulated kinase signaling regulates mTORC1 via TSC2 Ser664 (Ma et al., 2007). Total ERK protein was unchanged at 3 and 6 h in all groups (Figure 2A). ERK phosphorylation was decreased in 2B and 3B 3 h post‐RE. At 6 h after RE, 2B and 3B were lower than SED (Figure 2B). Total TSC2 protein decreased in the RE groups 6 h after RE (Figure 2E). Phosphorylation of TSC2 Ser664 decreased in the RE groups 3 h post‐RE and increased in the RE groups 6 h post‐RE (Figure 2F). Akt also regulates mTORC1 activity via TSC2 Ser939 and Thr1462 (Inoki et al., 2002). The Akt Ser473 phosphorylation levels increased in all the RE groups 3 h post‐RE, and only in 1B and 2B 6 h after RE, but not in 1B (Figure 2D). TSC2 Ser939 phosphorylation was increased in 1B and 3B and Thr1462 phosphorylation was increased in 2B and 3B 6 h after exercise (Figure 2G,H).

### 3.2 Downstream of mTORC1 signaling

**FIGURE 3:**
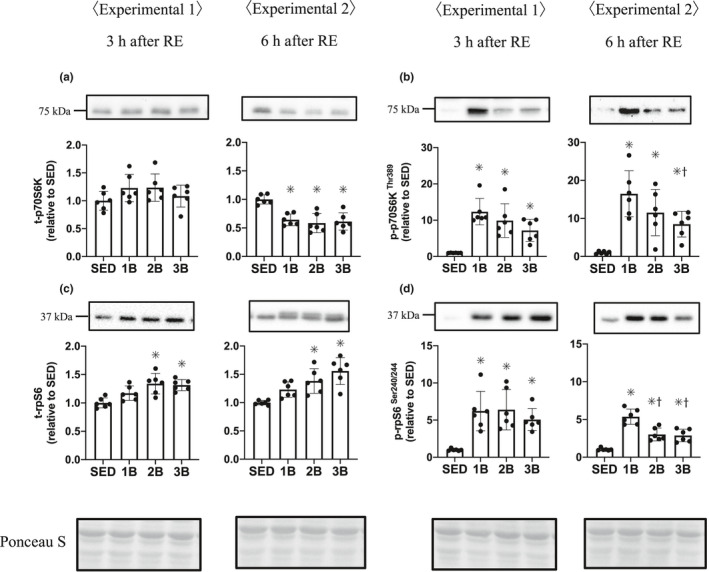
Effects of the number of resistance exercise (RE) bouts on p70S6K (a, b) and rpS6 (c, d). Protein phosphorylation and content are expressed relative to the mean value in the SED group. 1B, 2B, and 3B indicate resistance exercised with one bout, two bouts, and three bouts, respectively (*n* = 6 for each group). The timing of muscle sampling is denoted above each diagram. Values in the data are means ± SD. SED, sedentary for 5 days. *p < 0.05 versus SED; ^†^ *p* < 0.05 versus 1B

We measured the p70S6K Thr389 and rpS6 Ser240/244 phosphorylation levels as the mTORC1 downstream signaling indicators. The p70S6K Thr389 phosphorylation level was higher in all the RE groups 3 and 6 h post‐RE than in SED, with a significantly higher level in 1B than in 3B 6 h post‐RE (Figure 3B). In addition, the rpS6 Ser240/244 phosphorylation level increased from that in SED after RE, but the magnitude of increase was higher in 1B than in 2B and 3B 6 h post‐RE (Figure 3D).

### 3.3 Calpain pathway

**FIGURE 4:**
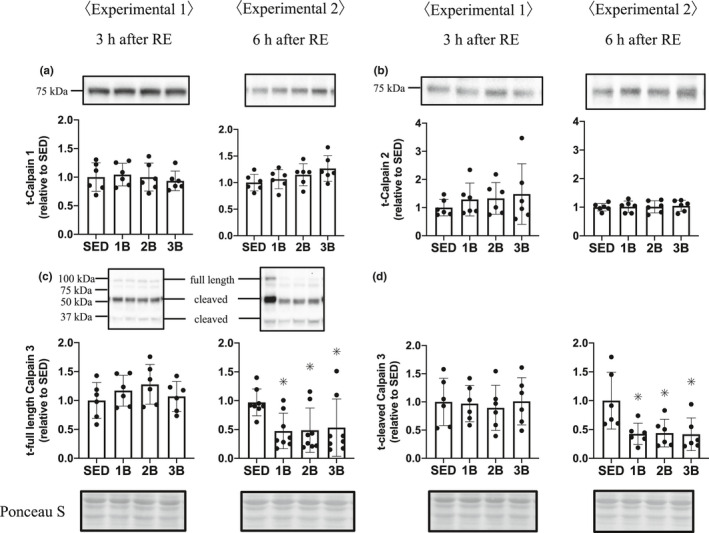
Effects of the number of resistance exercise (RE) bouts on calpain. Protein content per unit muscle weight is expressed relative to the mean value in the SED group. 1B, 2B, and 3B indicate resistance exercised with one bout, two bouts, and three bouts, respectively (*n* = 6 for each group). The timing of muscle sampling is denoted above each diagram. Values in the data are means ± SD. SED, sedentary for 5 days. *p < 0.05 versus SED

In the calpain pathway, calpain 1 and calpain 2 expressions were unchanged 3 and 6 h post‐RE in all exercised groups (Figure 4A,B). Calpain 3, which is a muscle‐specific member of the calpain subunit family that specifically binds to titin, was decreased in both full length and cleaved 6 h post‐RE in all exercised groups, regardless of the RE bout number (Figure 4C,D).

### 3.4 Autophagy–lysosome pathway

**FIGURE 5:**
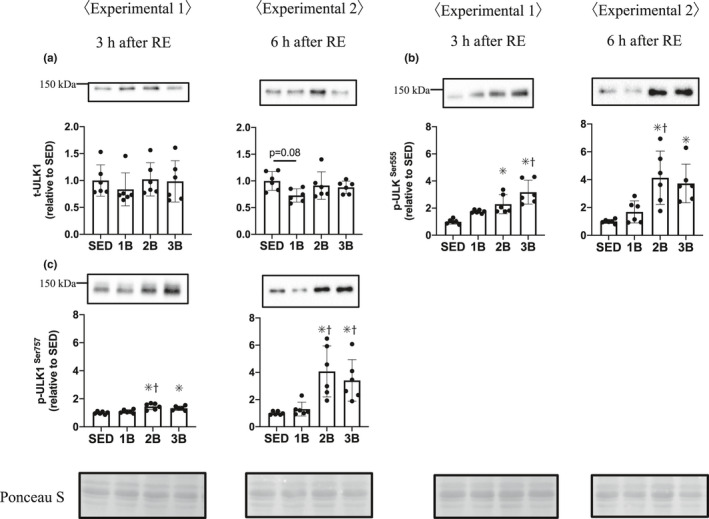
Effects of the number of resistance exercise (RE) bouts on UNC‐51‐like kinase (ULK1). Protein phosphorylation and content per unit muscle weight are expressed relative to the mean value in the SED group. 1B, 2B, and 3B indicate resistance exercised with one bout, two bouts, and three bouts, respectively (*n* = 6 for each group). The timing of muscle sampling is denoted above each diagram. Values in the data are means ± SD. SED, sedentary for 5 days. *p < 0.05 versus SED; ^†^ *p*<0.05 versus 1B

**FIGURE 6:**
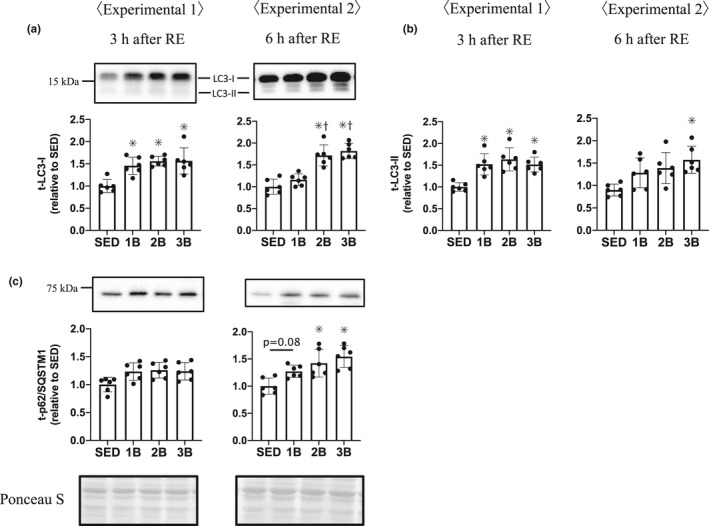
Effects of the number of resistance exercise (RE) bouts on LC3 (a, b) and p62/SQSTMI (c). Protein phosphorylation and content per unit muscle weight expressed relative to the mean value in the SED group. 1B, 2B, and 3B indicate resistance exercised with one bout, two bouts, and three bouts, respectively (n = 6 for each group). The timing of muscle sampling is denoted above each diagram. Values in the data are means ± SD. SED, sedentary for 5 days. *p < 0.05 versus SED

To examine the autophagy response, we measured the ULK1 phosphorylation level and LC3 and p62 protein content. The phosphorylation of ULK1 at Ser‐555, a positive regulator of autophagy, increased in 2B and 3B from that in SED, 3 and 6 h post‐RE (Figure 5B). In addition, the level of ULK1 Ser‐555 phosphorylation was higher in 3B than in 1B 3 h post‐RE and higher in 2B than in 1B 6 h post‐RE. The phosphorylation level of ULK1 at Ser‐757, an mTORC1 dependent negative regulator of autophagy, was also higher in 2B and 3B than in SED, 3 and 6 h post‐RE (Figure 5C). Furthermore, the level of ULK1 Ser‐757 phosphorylation was higher in 2B than in 1B 3 h post‐RE and was higher in 2B and 3B than in 1B 6 h post‐RE. LC3‐I and ‐II protein levels (autophagosome formation markers) were higher in all the RE groups than in SED 3 h post‐RE. LC3‐I increased in 2B and 3B than in SED and 1B, and LC3‐II increased in 3B than in SED 6 h post‐RE (Figure 6A,B). The level of p62 protein, a selective autophagy marker (Komatsu et al., 2007; Pankiv et al., 2007), was increased in 2B and 3B, compared with SED 6 h post‐RE (Figure 6C).

### 3.5 Ubiquitin–proteasome pathway

**FIGURE 7:**
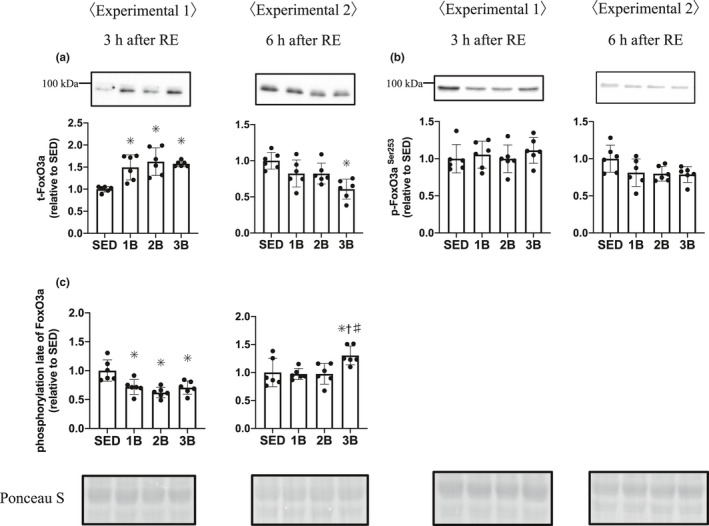
Effects of the number of RE bouts on FoxO3a. Protein phosphorylation, protein content, and phosphorylation late per unit muscle weight are expressed relative to the mean value in the SED group. (a), (b), and (c) are changes in muscle. 1B, 2B, and 3B indicate resistance exercised with 1 bout, 2 bouts, and 3 bouts, respectively (*n* = 6 for each group). The timing of muscle sampling is denoted above each diagram. Values in the data are means ± SD. SED, sedentary for 5 days. *p < 0.05 versus SED; ^†^ *p* < 0.05 versus 1B; ^#^ *p*<0.05 versus 2B

**FIGURE 8:**
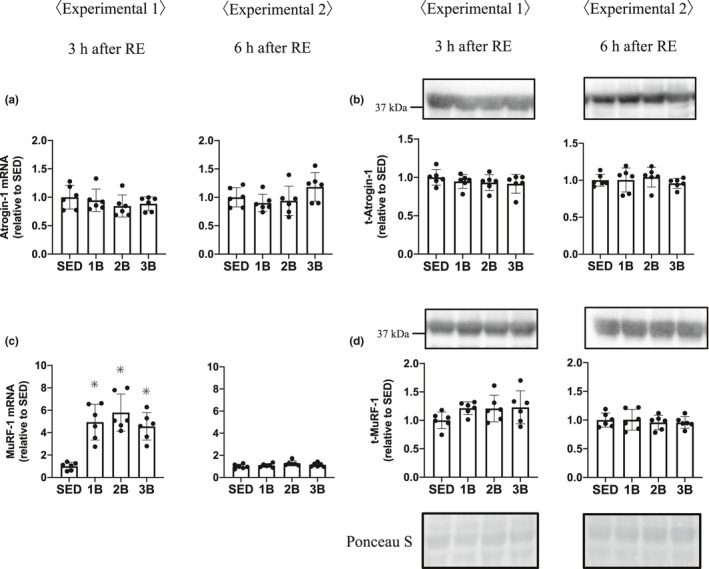
Effects of the number of resistance exercise (RE) bouts on Atrogin‐1 (a, b) and MuRF‐1(c, d). mRNA expression and protein content per unit muscle weight are expressed relative to the mean value in the SED group. 1B, 2B, and 3B indicate resistance exercised with one bout, two bouts, and three bouts, respectively (*n* = 6 for each group). The timing of muscle sampling is denoted above each diagram. Values in the data are means ± SD. SED, sedentary for 5 days. *p < 0.05 versus SED

**FIGURE 9:**
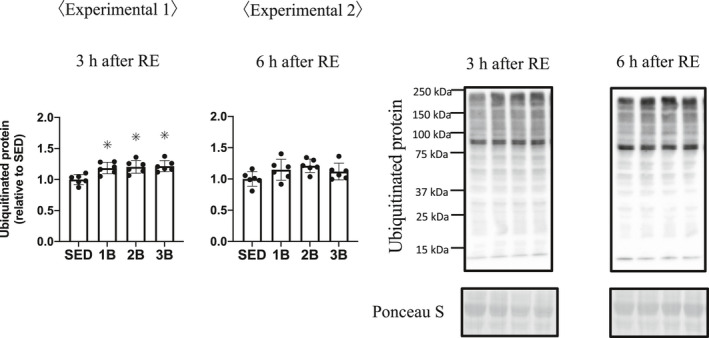
Effects of the number of resistance exercise (RE) bouts on ubiquitinated protein. The protein content per unit muscle weight is expressed relative to the mean value in the SED group. 1B, 2B, and 3B indicate resistance exercised with one bout, two bouts, and three bouts, respectively (*n* = 6 for each group). The timing of muscle sampling is denoted above each diagram. Values in the data are means ± SD. SED, sedentary for 5 days. *p < 0.05 versus SED

We measured the E3 ubiquitin ligases MuRF‐1 and atrogin‐1, and FoxO3a protein, a transcription factor of ubiquitin ligases. The total FoxO3a protein was increased in the RE groups 3 h post‐RE (Figure 7A); however, FoxO3a phosphorylation Ser253 did not change after RE (Figure 7B). FoxO3a is dephosphorylated and translocates into the nucleus, activating the initiation of transcription. We showed the phosphorylation rate of FoxO3a because of the increase in FoxO3a protein content. The phosphorylation rate of FoxO3a was decreased in the RE groups 3 h post‐RE (Figure 7C). These results mean that the dephosphorylated FoxO3a protein content is higher in the RE groups than in SED. At 6 h after RE, phosphorylation rate was higher in 3B than in SED, 1B and 3B. Atrogin‐1 mRNA expression and protein remained unchanged (Figure 8A,B). MuRF‐1 mRNA expression increased in all the RE groups, 3 h post‐RE (Figure 8C), but the MuRF‐1 protein did not change after RE (Figure 8D). The content of ubiquitinated proteins increased in all the RE groups 3 h post‐RE compared with SED (Figure 9).

## 4 DISCUSSION

The present study aimed to investigate the effects of increasing RE bouts on skeletal muscle proteolytic signaling responses when mTORC1 activation is physiologically attenuated in 3B of RE compared with 1B. The findings of this study were that (1) electrical stimulation‐induced RE increased LC3‐II (autophagosome formation markers) 3 h following exercise, regardless of RE bout number. However, at the 6 h time point when p70S6K activation was attenuated, an increase in LC3‐II was observed with increasing RE bouts. Secondly, (2) RE caused increases in the MuRF‐1 (ubiquitin ligase) mRNA expression and ubiquitinated proteins; however, additional bouts of RE did not affect the magnitude of these changes. These results suggest that repeated RE bouts do not affect the degree of activation of the ubiquitin–proteasome system, but affect the autophagy system.

Resistance exercise activates both the anabolic and catabolic responses in skeletal muscle (Phillips et al., 1997). mTORC1 signaling is one of the major regulators of muscle protein synthesis and is activated by RE (Baar & Esser, 1999; Ogasawara et al., 2016; West et al., 2016). Our study showed the activation of p70S6K and rpS6, which are downstream targets of mTORC1 signaling; it also suggests activated muscle protein synthesis similar to those reported in previous studies. Repeated bouts of RE reduce the activation of mTORC1 signaling downstream targets p70S6K and rpS6 (Coffey et al., 2005; Ogasawara et al., 2013). In agreement with these findings, we observed that the phosphorylation level of p70S6K and rpS6 was lower in 3B than in 1B at 6 h post‐RE. ERK1/2 and Akt regulate mTORC1 activity via TSC2 inhibition or regulatory‐associated proteins of mTOR phosphorylation. Previous studies showed that ERK1/2 and Akt are phosphorylated following RE (Ato et al., 2017; Nader, & Esser, 2001; Ogasawara et al., 2017). However, we did not observe the phosphorylation of ERK. Previous studies reported that ERK is phosphorylated immediately after RE (Ato et al., 2017; Nader & Esser, 2001). In addition, Franchi et al. reported that eccentric loading activates ERK more than concentric loading (Franchi et al., 2014). From these findings, it is possible that phosphorylation of ERK could not be observed in this study due to differences in intensity and/or time points. Conversely, ERK phosphorylation was decreased in 2B and 3B, which might be induced by negative regulatory factors on ERK by repeated bouts of RE. However, TSC2 Ser664 (downstream of ERK1/2) was phosphorylated 6 h post‐RE in all exercised groups, regardless of the number of RE bouts. We hypothesized that the ERK‐TSC2 pathway would not be associated with the attenuation of RE‐induced mTORC1 activation after RE. Next, we investigated the Akt activity, which was observed to diminish the degree of exercise‐induced phosphorylation 6 h post‐RE in 3B. Akt regulates mTORC1 through the phosphorylation of TSC2 Ser939 and Thr1462. In the present study, RE induced the phosphorylation of both sites, but there was no considerable difference between the magnitudes of changes. Repeated bouts of RE attenuated the activation of mTORC1, but this was not due to the upstream factors investigated in this study and should be further investigated in future studies.

It has been shown that major proteolytic systems play important roles in the breakdown of muscle proteins: the autophagy–lysosome system and the ubiquitin–proteasome system (Sandri, 2013). mTORC1 signaling downregulates the autophagy response via ULK1 Ser‐757 phosphorylation (Kim et al., 2011), and RE causes the phosphorylation of ULK1 Ser‐757 (Ato et al., 2017; Ogasawara et al., 2016). In agreement with these studies, the phosphorylation level of ULK1 Ser‐757 was elevated in 2B and 3B. ULK1 Ser‐757 phosphorylation did not decrease with the attenuation of p70S6K activity but increased at 6 h post‐RE. Ogasawara et al. reported that the ULK1 Ser‐757 phosphorylation level was elevated after RE even if rapamycin was added to suppress the mTORC1 signaling activity in the rat RE model (Ogasawara et al., 2016). These findings suggest that the phosphorylation of ULK1 Ser‐757 after RE is also induced by the mTORC1 signaling‐independent pathway. ULK1 Ser‐555 phosphorylation, which activates autophagy, increased in 2B and 3B. In previous studies of the activation of autophagy with overtraining or denervated‐induced muscle atrophy, both ULK1 Ser757 and Ser555 were phosphorylated, and LC3‐II was increased (Takegaki et al., 2019; Tamura et al., 2015). Therefore, we suggested that repeated bouts of RE activated autophagosome formation via ULK1 phosphorylation. We subsequently focused on LC3 as a major indicator of autophagy. The autophagy system is initiated by several factors and LC3 is produced as a propeptide, then is immediately cleaved, and the peptide containing C‐terminus is released into the cytoplasm as LC3‐I. Phosphatidylethanolamine is added to the C‐terminus of LC3‐I to form LC3‐II. LC3‐II is incorporated into the isolation membrane, to which it strongly binds. Thus, LC3‐II is commonly used as a marker of autophagosome formation (Kabeya et al., 2000). Earlier studies have shown that LC3‐II is decreased or unchanged with RE in humans and rats (Fry et al., 2013; Ogasawara et al., 2016; Pankiv et al., 2007). However, we observed that LC3‐II levels were higher in all the RE groups 3 h post‐RE. One of the reasons for the discrepancies with previous LC3‐II results may be the differences in the nutritional conditions before RE. Fasting is known to increase LC3‐II levels in rodent and human skeletal muscles (Jamart et al., 2013; Mizushima et al., 2004; Vendelbo et al., 2014). In the present study, animals were not fasted before RE. In previous studies, fasting before RE might have activated autophagy and masked the effects of RE. The present results showed that RE increases the LC3‐II protein content in all RE groups 3 h post‐RE, and only 3B showed an increase in LC3‐II 6 h post‐RE. LC3‐I also was increased 6 h post‐RE with increasing bouts of RE. p62, which has been shown to be involved in selective autophagy by interacting with LC3 (Komatsu et al., 2007; Komatsu et al., 2007; Pankiv et al., 2007), was increased in 2B and 3B 6 h post‐RE. The data generated in the present study of ULK1, LC3, and p62 proteins suggest that increasing bouts of RE accelerate autophagosome formation when mTORC1 activation is attenuated with increasing RE bouts.

The ubiquitin–proteasome system also regulates muscle protein breakdown (Bodine & Baehr, 2014; Bodine, Latres, et al., 2001; Bodine, Stitt, et al., 2001). Ubiquitination is mediated by ubiquitin ligase, such as MuRF‐1 or atrogin‐1, and it is recognized that the expression of these ubiquitin ligases is regulated by the Akt‐ FoxO3a pathway (Sandri et al., 2004). FoxO3a is dephosphorylated and translocates from the cytoplasm to the nucleus and promotes the transcription of the ubiquitin ligases gene. In the present study, the total FoxO3a proteins were increased, and the rate of phosphorylation (phospho/total of FoxO3a) was decreased. A previous study showed that FoxO3a‐knockdown prevents dexamethasone‐induced MuRF‐1 expression (Kang et al., 2017). We observed an increase in only MuRF‐1 mRNA expression following RE. Previous studies have also shown that acute RE increased the expression of MuRF‐1 mRNA, but not that of atrogin mRNA (Fry et al., 2013; Glynn et al., 2010). In addition, the present study showed that ubiquitinated proteins were increased 3 h post‐RE. MuRF‐1 null mice attenuate the increase in ubiquitin and skeletal muscle atrophy by dexamethasone (Baehr et al., 2011). Based on these data, we suggest that RE increased MuRF‐1 expression through FoxO3a and promoted the ubiquitination of muscle protein in the present study.

The present results suggest that RE increased the dephosphorylated FoxO3a protein. Although the dephosphorylation of FoxO3a increased, we only observed an increase in MuRF‐1 mRNA following RE. However, protein levels of MuRF‐1 were not increased, in contrast to the increase with approximately four–five‐folds’ change of MuRF‐1 mRNA expression. We suggest that the MuRF‐1 protein increased following exercise, but we were unable to observe the increase in MuRF‐1 protein because of some reasons (time course or antibody specificity, etc.). Ubiquitinated proteins also were increased 3 h post‐RE. These results suggest that acute RE dephosphorylated FoxO3a and promoted the transcription of MuRF‐1 and the ubiquitination of muscle protein. However, the present study showed that repeated RE does not attenuate RE‐induced increases in MuRF‐1mRNA expression, FoxO3a dephosphorylation, and ubiquitinated proteins.

## 5 CONCLUSION

In conclusion, repeated bouts of RE did not change the degree of increase in ubiquitin ligase expression and ubiquitination, but they increased autophagosome formation when mTORC1 activity was attenuated.

## CONFLICT OF INTEREST

The authors declare that they have no competing interests.

## ETHICS APPROVAL AND CONSENT MATERIALS

The present study was approved by the Animal Ethics and Research Committee of The University of Tokyo (no. 27‐13), and all experiments followed the guidelines of the committee.

## CONSENT FOR PUBLICATION

Not applicable.

## AUTHOR CONTRIBUTION

Takaya Kotani, Junya Takegaki, and Naokata Ishii conceived and designed research. Takaya Kotani performed experiments and analyzed data. Takaya Kotani, Junya Takegaki, Yuki Tamura, Karina Kouzaki, Koichi Nakazato, and Naokata Ishii interpreted results of experiments. Takaya Kotani prepared figures and drafted manuscript. All authors read and approved the final manuscript, and agree to be accountable for all aspects of the work in ensuring that questions related to the accuracy or integrity of any part of the work are appropriately investigated and resolved. All persons designated as authors qualify for authorship, and all those who qualify for authorship are listed.

## Data Availability

The datasets used and/or analyzed during the current study are available from the corresponding author on reasonable request.
